# Evaluation of a novel simulation method of teaching B-lines: hand ultrasound with a wet foam dressing material

**DOI:** 10.1186/2036-7902-7-S1-A30

**Published:** 2015-03-09

**Authors:** YJ Cho, KH Lee, JH Ahn, CK Hong, YR Ha

**Affiliations:** 1 Department of Internal Medicine, Seoul National University Bundang Hospital, South Korea; 2 Department of Emergency Medicine, Bundang Jesaeng Hospital, South Korea

**Keywords:** Simulated Model, Interventional Radiology, Correct Answer, Simulation Method, Video Clip

## Background

Lung ultrasound simulations for pathologic conditions are not readily available for bedside teaching. Recently hand ultrasound was introduced as a new model of simulating lung ultrasound including normal lung sliding, stratosphere sign, and lung point. However no effective method of teaching B-lines has been reported.

## Objective

The aim of this study was to evaluate effectiveness of a novel mode of teaching B-lines made by using hand ultrasound with a wet foam dressing material simulating a wet lung.

## Patients and methods

All subjects enrolled were medical school students who were novice for lung ultrasound. All subjects attended a 20-mintutes lecture about lung ultrasound using simulated video clips of A-lines, B-lines, and lung sliding for 20 minutes and 20-minutes post-test was given. A post-test were composed of questions on the choice between A-lines and B-lines and the presence of lung sliding using randomly mixed 20 real and 20 simulated video clips using hand ultrasound with or without a wet foam dressing materials. At the end of the post-test, the correct answer was revealed and discussed. Paired t test was used to compare the each score of A-lines, B-lines, and lung sliding between the real images and simulated models.

## Results

There were 56 male and 20 female with mean age of 25.1±2.8. The mean of the total score was 51.9±4.9 for the real video clips and 52.3±5.0 for the simulated models (P=0.485). The mean of the score for correct answers between A-lines and B-lines was 17.5±2.6 for the real video clips and 17.0±2.0 for the simulated clips (P=0.0961) The mean of the score for lung sliding was 16.0±2.7 in real image and 17.6±2.6 in simulated images (P<0.001).

## Conclusion

The novel B-line teaching model by using a hand ultrasound with a wet foam dressing material was readily available and effective method to simulate pulmonary interstitial syndrome.

## References

[1] Shokoohi H, Boniface K (2012). Hand Ultrasound: A High-fidelity Simulation of Lung Sliding. Acad Emerg Med.

